# Effectiveness of Computerized Cognitive Training in Delaying Cognitive Function Decline in People With Mild Cognitive Impairment: Systematic Review and Meta-analysis

**DOI:** 10.2196/38624

**Published:** 2022-10-27

**Authors:** Ran Li, Jiawei Geng, Runze Yang, Yumeng Ge, Therese Hesketh

**Affiliations:** 1 Institute for Global Health University College London London United Kingdom; 2 Centre for Global Health School of Public Health Zhejiang University Hangzhou Zhejiang China

**Keywords:** computerized cognitive training, mild cognitive impairment

## Abstract

**Background:**

With no current cure for mild cognitive impairment (MCI), delaying its progression could significantly reduce the disease burden and improve the quality of life for patients with MCI. Computerized cognitive training (CCT) has recently become a potential instrument for improvement of cognition. However, the evidence for its effectiveness remains limited.

**Objective:**

This systematic review aims to (1) analyze the efficacy of CCT on cognitive impairment or cognitive decline in patients with MCI and (2) analyze the relationship between the characteristics of CCT interventions and cognition-related health outcomes.

**Methods:**

A systematic search was performed using MEDLINE, Cochrane, Embase, Web of Science, and Google Scholar. Full texts of randomized controlled trials of CCT interventions in adults with MCI and published in English language journals between 2010 and 2021 were included. Overall global cognitive function and domain-specific cognition were pooled using a random-effects model. Sensitivity analyses were performed to determine the reasons for heterogeneity and to test the robustness of the results. Subgroup analyses were performed to identify the relationship between the characteristics of CCT interventions and cognition-related effectiveness.

**Results:**

A total of 18 studies with 1059 participants were included in this review. According to the meta-analysis, CCT intervention provided a significant but small increase in global cognitive function compared to that in the global cognitive function of the control groups (standardized mean difference=0.54, 95% CI 0.35-0.73; *I*^2^=38%). CCT intervention also resulted in a marginal improvement in domain-specific cognition compared to that in the control groups, with moderate heterogeneity. Subgroup analyses showed consistent improvement in global cognitive behavior in the CCT intervention groups.

**Conclusions:**

This systematic review suggests that CCT interventions could improve global cognitive function in patients with MCI. Considering the relatively small sample size and the short treatment duration in all the included studies, more comprehensive trials are needed to quantify both the impact of CCT on cognitive decline, especially in the longer term, and to establish whether CCT should be recommended for use in clinical practice.

**Trial Registration:**

PROSPERO International Prospective Register of Systematic Reviews CRD42021278884; https://www.crd.york.ac.uk/prospero/display_record.php?RecordID=278884

## Introduction

### Mild Cognitive Impairment

The older adult population is increasing worldwide. In 2017, 962 million people or 13% of the global population were over 60 years of age, and this figure is predicted to rise to 1.4 billion by 2030 [1]. This raises concerns about the growing global burden of degenerative disorders, especially dementia. The development of interventions to prevent, delay, and treat dementia is now recognized as a matter of urgency [2]. Mild cognitive impairment (MCI) is recognized as an intermediary phase between the cognitive changes of normal aging and the onset of dementia, suggesting that it may represent an opportune time to prevent or delay the onset of dementia. Petersen et al [3] reported that globally, an estimated 14.9% of people with MCI aged over 60 years progressed to dementia in the following 2 years and one-third of people living with MCI develop dementia within 5 years [4]. The prevalence of MCI is estimated to be around 16% in adults aged over 60 years, with the risk increasing with age [2]. The diagnostic criteria for MCI include a change in cognition, abnormal cognitive function in one or more domains, but without notable interference in everyday functioning [5].

Currently, there is no specific diagnostic test for MCI. However, global cognitive function is measured most commonly using the Mini-Mental State Examination and the Montreal Cognitive Assessment [6]. Measures such as executive function, working memory, episodic memory, and quality of life are also commonly used. In this context, executive function is the most complex cognitive process necessary for goal-directed behavior [7]. Working memory is a limited capacity system that briefly stores and manages the information required in other cognitive operations [8]. Episodic memory is a past-oriented memory system that encodes, stores, and searches personally experienced events [9].

### Existing Interventions for MCI

The increasing prevalence of MCI and the risk of progression to dementia raises questions about interventions that delay or prevent this process [10]. Interventions for MCI can be divided into pharmacological interventions and nonpharmacological interventions. Currently, there are no specific pharmacological interventions for the treatment of MCI. In the United Kingdom, the 2 drugs used for Alzheimer disease, cholinesterase inhibitors and Memantine, have not been shown to help people with MCI [11]. The US Food and Drug Administration has given the drug Aducanumab accelerated approval as a treatment for Alzheimer disease and MCI. However, there is no evidence for the drug’s effectiveness data in the treatment of MCI [12]. Most nondrug interventions for MCI address the underlying modifiable causes of MCI, including lifestyle and the treatment of health conditions such as hypertension, obesity, diabetes, stroke, and vitamin deficiency [13]. However, there is no evidence for the effectiveness of dietary changes, including Vitamin E supplements, for delaying MCI [14]. Physical exercise programs have been shown to reduce a person’s risk of MCI development [15]. However, the effectiveness of increased physical activity in delaying or delaying the progress of cognitive disorders remains unclear [16]. Other nondrug interventions for MCI include memory training, staying mentally and socially active, and cognitive training [17]. The quality of interventions involving social activities for alleviating MCI remains controversial across existing studies [18,19].

### Noncomputerized and Computerized Cognitive Training

With insufficient evidence to support the use of pharmacological and nonpharmacological interventions as described above, cognitive training has been proposed as an intervention to improve cognitive function. This involves repeated activities based on the theory of brain plasticity [20]. With advances in computing technology, traditional cognitive training based on pen and paper has gradually been replaced by computerized cognitive training (CCT) in settings where there is good access to appropriate technology among target groups. CCT is an application of digital health in which individuals can access engaging and interactive cognitive exercises from their own computers, tablets, virtual reality (VR), or mobile devices [21]. CCT involves guided drill-and-practice on standardized tasks, typically without explicit teaching of memory or problem-solving strategies, which distinguish CCT from other approaches for cognitive training [22]. Compared with non-CCT, CCT is more accessible, comprehensive, and flexible to adaptation to individuals’ capacity. The game-like nature is often experienced as intrinsically rewarding [23]. In addition, CCT has generated considerable attention as a safe, relatively inexpensive, and scalable intervention that may maintain cognition in older adults [24]. Further, with enjoyable activities, immediate feedback, and automatic adaptations based on participants’ performance, CCT is thought to increase participants’ motivation and adherence [25].

### Existing Studies and Research Gap

Since 2010, a rapidly increasing number of studies started to evaluate the effectiveness of CCT programs specifically targeting certain cognitive domains such as memory [26], executive function, and processing speed [27]. Among them, working memory has garnered particular attention in recent years. A recent systematic review of the effectiveness of CCT has found moderate effect sizes on cognition in healthy older adults [28]. However, the effectiveness of CCT in addressing cognitive decline in people with MCI remains inconclusive. Most of the existing reviews, which synthesized evidences from randomized controlled trials (RCTs) of CCT on participants with MCI, revealed small-to-moderate effects on improving cognitive function [29-32]. Three reviews combined CCT and non-CCT therapies (such as therapeutic drugs, diet modification, and physical activity), providing conclusions about the specific effectiveness of CCT [29,31,32]. A recent Cochrane review included only interventions that lasted more than 12 weeks [33], but that review found only 8 studies with small sample sizes; therefore, conclusions about intervention effectiveness could not be drawn. Considering the rapid development and increasing accessibility of CCT in the last decade, updating the latest evidence about CCT is necessary to inform clinical practice. Therefore, we conducted this review to determine whether CCT is an effective intervention for addressing cognitive decline in people with MCI. The objectives of this review were to (1) analyze the effectiveness of CCT on preventing progression in cognitive decline and (2) explore the relationship between the characteristics of CCT interventions and cognition-related health outcomes.

## Methods

### Data Sources and Search Strategy

This systematic review and meta-analysis were performed according to PRISMA (Preferred Reporting Items for Systematic Reviews and Meta-Analyses) statement and was registered with PROSPERO (Prospective Register of Systematic Reviews; CRD42021278884). Five web-based databases, that is, MEDLINE, Cochrane, Embase, Web of Science, and Google Scholar were searched and updated in August 2021. The literature search used a combination of search terms and keywords for the following main concepts: “cognitive decline,” “mild cognitive impairment,” “cognitive training,” “cognitive exercise,” “computerized cognitive training,” “virtual reality,” and “technology.” All keywords were concatenated using Boolean operators and appropriate truncation symbols depending on database requirements. The detailed search strategy is shown in Multimedia Appendix 1 [25,34-50]. Snowballing methods identified potential papers by screening reference lists from relevant reviews.

### Eligibility Criteria

The inclusion and exclusion criteria were identified based on the PICO (Population, Intervention, Comparison, and Outcomes) approach as follows.

1. Study design: only full-text peer-reviewed RCTs published in English between 2010 and 2021 were included. Pilot studies and studies with abstract only were removed.
2. Population: The population of interest was adults aged 18 years or older who had MCI. Studies including healthy people or those already diagnosed with dementia or with other neurological and psychological disorders were excluded.
3. Intervention: Participants in the experimental groups were treated with CCT only. Studies in which CCT was used along with other therapies or drugs aiming to improve participants’ cognitive functions were removed. The programs used computers, consoles, and VR.
4. Control: Either active control (such as watching general education material and any non–CCT-based training) or usual care (without any intervention applied or waiting list) was included.
5. Outcomes: These included (1) participants’ global cognitive function; (2) specific cognitive function, including executive function, working memory, and episodic memory; and (3) new cases of dementia.

### Study Selection and Data Extraction

Two reviewers (RL and RY) independently conducted the initial search of the databases by looking through titles and abstracts. Then, the full text of the included studies was reviewed against the eligibility criteria. The snowballing method was used for the reference lists of the relevant papers. Study citations were imported into the reference management software (Endnote X8.0, Clarivate Analytics) for selection. Any disagreement was resolved by discussing with an additional reviewer.

Three authors extracted the following data: (1) study characteristics (author, year of publication, study location), (2) information of participants (study population, number of patients, gender, age), (3) details about activities in intervention and control group (duration of intervention, frequency of intervention, time per session, delivery device, feedback providing mechanism, interactive patterns, and activities), (4) relevant cognitive function outcomes, including global and specific cognitive function, and (5) when outcomes were measured at multiple time points, measures immediately after the completion of the intervention were extracted. All data were checked by an independent researcher (RL).

### Data Synthesis and Analysis

The primary outcome of this review was participants’ global cognitive function, which assessed individuals’ general cognitive status. Secondary outcomes were domain-specific cognitive function, including executive function associated with goal-directed behavior [7]; working memory regarding attentional and short-term memory [8]; episodic memory or long-term memory that encodes, stores, and searches personally experienced events [9]; visual memory; and verbal memory. The R software (R Core Team and the R Foundation for Statistical Computing; version 4.1.2) was used to analyze the quantitative data, and a two-tailed *P* value of less than .05 was defined as statistically significant. As all the outcomes of effectiveness of CCT were continuous variables, standardized mean differences (SMDs) estimated by Hedge’s g method and their corresponding 95% CIs were used to determine the effect size based on the differences between preintervention and postintervention. For studies with multiple interventions, we calculated the effect size separately for each comparison. Due to the possibility of between-study heterogeneity, the random-effects model was used in the meta-analysis with the pooling method of DerSimonian-Laird. Heterogeneity was evaluated by *χ*^2^ (Cochrane Q), *I*^2^, and *Tau*^2^ statistics and displayed in forest plots. To quantify the magnitude of heterogeneity, we defined a value of *I*^2^ more than 50% as moderate-to-high heterogeneity. Funnel plots were applied to assess publication bias if more than 10 papers were available for an outcome in the meta-analysis. Besides visual inspection, Egger and Begg tests were conducted to adjust the potential effect of publication bias on the interpretation of the results [51]. Furthermore, to test the robustness of the results, sensitivity analyses were conducted using the leave-one-out method. To explore the effects of different characteristics of patients and CCT interventions on the impact of measured effectiveness of global cognitive function, we conducted prespecified subgroup analyses by testing 1 variable at a time. Intervention characteristics included year of publication, delivery devices (computer/tablet or other technology), CCT-targeted domains (multiple or single), feedback provided after treatment or not, interactive patterns (interventions with a patient-provider discussion after treatment), intervention settings (intervention carried out in a group or an individual), and training dose with cutoff chosen at mean values, including duration (less than 3 months or not), frequency (less than 3 days per week), and time per intervention session (less than 1 hour). Comparator characteristics were defined as whether patterns of activities were actively controlled or passively controlled.

### Assessment of Risk of Bias

To adequately assess the risk of bias (ROB) in the included studies in this review, the Cochrane ROB tool was used (version 5.4). All information about the features of the process of randomization, allocation concealment, blinding of participants, blinding of outcome assessors, incomplete outcome, and selective reporting were assessed. In addition, the risk of funding bias and baseline imbalance were considered. The ROBs in this review were classified as “high ROB,” “low ROB,” or “unclear ROB.”

## Results

### Search Results

**Figure 1:**
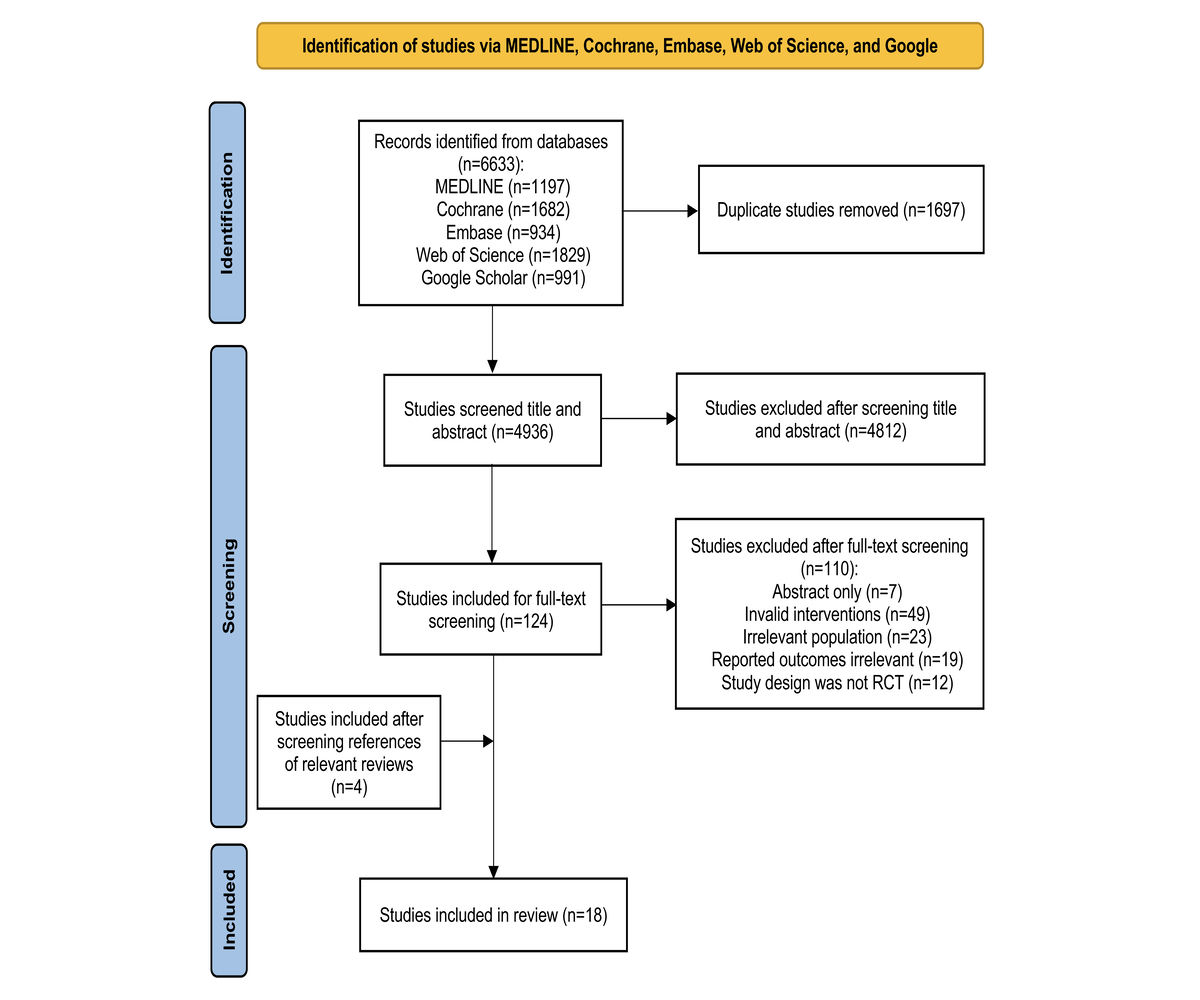
Flow diagram of study selection. RCT: randomized controlled trial.

As shown in Figure 1, the initial search found 4936 studies after excluding 1697 duplicated records. A further 4812 records were excluded after screening the titles and abstracts of the remaining records. A total of 124 full‐text records were assessed for eligibility and 110 records were further excluded. Of these, 7 studies were English abstracts only, 49 studies had invalid interventions (such as the treatment was not CCT or the control group received other interventions with treatment effects), 19 studies reported outcomes irrelevant to the aims of this review (such as safety, acceptance, and feasibility of CCT), 23 studies had irrelevant populations (such as healthy older people and people with dementia), and the study designs of 12 studies were not RCTs. An additional 4 studies were identified from references of relevant reviews. After the above selection process, 18 studies were included in this review.

### Study Characteristics

The characteristics of the participants and trials in the included studies are shown in Table 1. A total of 18 different RCTs with 1059 participants were published between January 2010 and August 2021, 8 of which were published since 2019. Sample sizes ranged from 22 to 141, and the mean age of the participants ranged from 58.8 years to 78.2 years. All studies were conducted in high-and-middle-income countries.

**Table 1:** Study characteristics.

Study	N^a^	Mean (SD) age (years)	Intervention characteristics	Control group
Liao et al [34], 2020	21/21	73.1 (6.8)	VR^b^: physical activity + cognitive training	Active: combined physical and cognitive training but not CCT^c^-based (reciting poems, crossing obstacles practicing math calculations, etc)
Thapa et al [35], 2020	33/33	72.6 (5.4)	VR: physical activity + cognitive training	Active: educational program on general health care
Park [44], 2020	28/28	71.9 (3.1)	VR: physical activity designed to improve spatial memory	Passive: did not engage in any activity
Park et al [25], 2020	18/17	75.8 (8.5)	VR: spatial cognitive task	Active: tabletop activities, maze and pencil-paper with table activities
Li et al [43], 2019	78/63	69.5 (7.3)	Computer: cognitive training	Passive: not provided with cognitive intervention
Nousia et al [48], 2019	25/21	71.2 (5.1)	Computer: cognitive rehabilitation	Passive: standard clinical care
Yang et al [36], 2019	33/33	75.4 (6.6)	VR: working memory training	Active: reading web-based e-books and playing web-based games such as puzzles
Oh et al [49], 2018	37/16	58.8 (5.0)	Smartphone: cognitive training	Passive: wait-list
Pereira-Morales et al [46], 2017	12/11	64.5 (4.8)	Web application: cognitive training	Active: received only an information brochure to read at home
Savulich et al [37], 2017	21/21	75.2 (7.4)	iPad game: cognitive training	Passive: clinic as usual
Han et al [47], 2017	43/42	73.7 (4.8)	iPad tablet: cognitive training	Passive: usual care
Hyer et al [50], 2016	34/34	75.1 (7.4)	Computer: working memory training	Active: sham cognitive training
Gooding et al [41], 2016 (CCT), and Gooding et al [41], 2016 (CVT^d^)	31/20, 23/20	75.6 (8.8)	Computer: plasticity-based training program; Computer: traditional CCT that is embedded within Neuropsychological and Educational Approach to Remediation model of treatment	Active: computer games and puzzles
Barban et al [39], 2016	46/60	74.4 (5.7)	Computer: reminiscence therapy + cognitive training	Passive: cross-over (rest)
Styliadis et al [42], 2015	14/14	67.6 (4.0)	Computer: cognitive training	Active: underwent a training protocol consisting of watching a documentary and answering questionnaire
Fiatarone Singh et al [40], 2014	22/24	70.1 (6.7)	Computer: cognitive training + sham exercise	Active: sham cognitive (watch short videos) + sham exercise (stretching and seated calisthenics)
Bozoki et al [45], 2013	32/28	68.9 (6.8)	Computer: cognitive training + mental training	Active: thoughts in motion; sound thinking; headline clues
Herrera et al [38], 2012	11/11	78.2 (1.4)	Computer: memory and attention training	Active: cognitive activities including find names of countries and corresponding capitals etc

^a^Number of participants in intervention group/control group.

^b^VR: virtual reality.

^c^CCT: computerized cognitive training.

^d^CVT: cognitive vitality training.

### CCT Characteristics

Common activities included attention training, visual processing, sensory integration, and recollection exercises. Thirteen studies were delivered as cognitive training programs on computers or tablets [37-43,45-50]. Another 5 studies [25,34-36,44] used VR-based interactive video games, with 1 study combining both tablets and VR devices [37]. In 5 studies, participants completed all treatment in groups under supervision by trained cognitive therapists [27,37-40]. Others carried out CCT interventions by themselves. The frequency of CCT sessions was 2-5 times per week, with a mean frequency of 3 times per week. The length of each session was around an hour in all 18 studies. Mean trial duration was 10.5 (range 4-24) weeks. The average dropout rate in the studies was 8% (range 0%-23%). The main reasons for dropout were unwillingness to continue and unrelated health issues. Eight studies reported no missing data from baseline to completion [25,36-38,42,44,46,48]. The activities of the CCT programs were diverse and 7 of them targeted multidomain cognitive function [34,35,39-43]. Most CCT programs included more than 1 activity, including remembering items in a limited time, mathematical calculations, and auditory stimuli (an auditory stimulus and recognizing a synthetically generated syllable from a confusable pair). CCT interventions in some studies, especially VR-based CCT interventions, inevitably combined some physical activities [34,35,39] such as balance training, agility training, strength training, and flexibility training. In 7 studies [25,34,37,41,44-46], feedback was provided to the participants, either in real time or as they finished each activity during the CCT session, such as “Good job,” “Better next time,” and visual and auditory feedback. Seven studies conducted interactions between providers and patients in CCT groups during the intervention or after they finished each session [25,35,38-40,46,47].

### Outcome Measures

#### Global Cognitive Function

**Figure 2:**
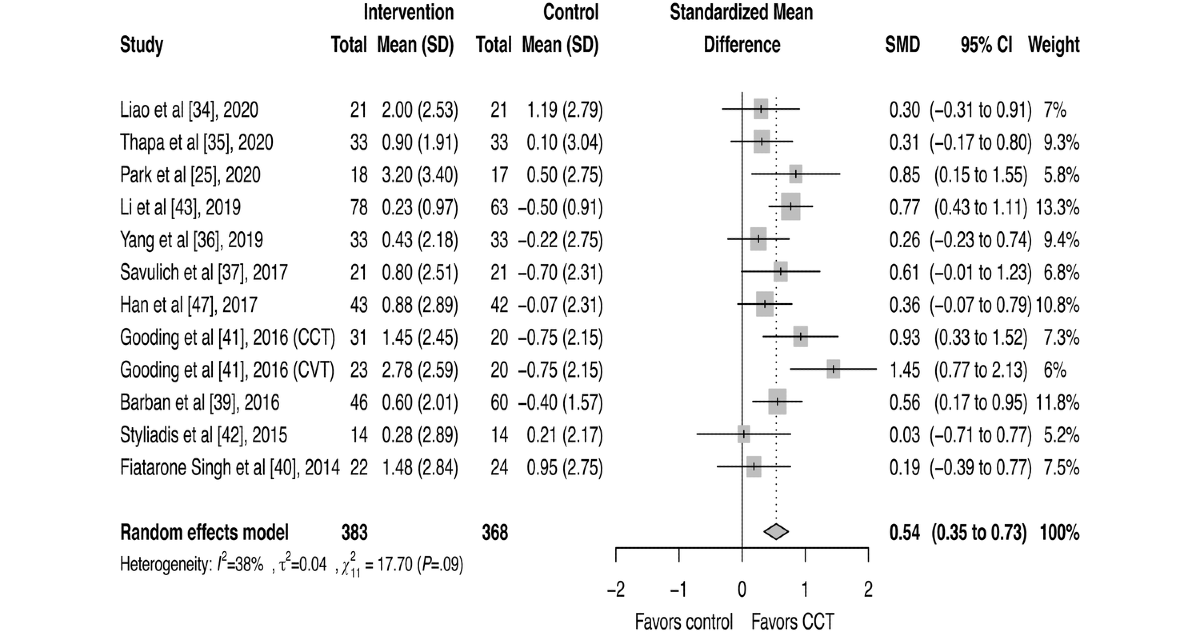
Forest plot for global cognitive function [25,34-37,39-43,47]. CCT: computerized cognitive training; CVT: cognitive vitality training; SMD: standardized mean difference.

Eleven studies measured the change in global cognitive function between preintervention and postintervention immediately after completion of the whole treatment by using Mini-Mental State Examination [35-37,39,41-43,47], Montreal Cognitive Assessment [25,34], or Alzheimer’s Disease Assessment Scale-Cognitive subscale [40]. Gooding et al [41] had more than one intervention group with the same outcomes measured. Therefore, 12 trials were shown in the meta-analysis of global cognitive function. The pooled SMD of global cognitive function (Figure 2) showed a statistically significant improvement for participants in the intervention groups compared to that in the control groups (SMD=0.54, 95% CI 0.35-0.73), with moderate heterogeneity between studies (*P*=.09; *I^2^*=38%). No significant publication bias was suggested, as no asymmetry was detected in the funnel plot (Multimedia Appendix 2), and neither Egger (*P*=.98) nor Begg (*P*=.89) tests were significant. Effect size in sensitivity analysis remained significant with no notable change (Multimedia Appendix 3 [25,34-37,39-43,47]). Subgroup analyses (Table 2) showed consistent improvement in global cognitive behavior in the CCT intervention groups across all variables mentioned above. However, we observed no significant difference in the effect size in each comparison.

**Table 2:** Subgroup analyses of global cognitive function.

Study subgroup characteristic				Trials (n)		*I*^2^ (%)		Standardized mean difference (95% CI)		*P* value
Overall				12		38		0.54 (0.35-0.73)		N/A^a^
**Intervention characteristics**										
	**Publication^b^**									.75
		2019 or later	5		23		0.51 (0.26-0.76)			
		Prior to 2019	7		52		0.58 (0.27-0.88)			
	**Delivery devices**									.22
		Computer/tablets	8		48		0.61 (0.35-0.86)			
		Virtual reality	4		0		0.38 (0.10-0.65)			
	**Computerized cognitive training type**									.51
		Multidomain	8		52		0.57 (0.30-0.84)			
		Single domain	4		0		0.45 (0.18- 0.71)			
	**Interaction**									.33
		With	5		0		0.43 (0.22-0.65)			
		Without	7		54		0.62 (0.31-0.93)			
	**Feedback**									.29
		With	3		67		0.85 (0.19-1.52)			
		Without	9		13		0.49 (0.31-0.66)			
	**Setting**									.96
		Activities in group with supervision	4		0		0.53 (0.27-0.80)			
		Individual	8		55		0.54 (0.27-0.82)			
**Training dose**										
	**Duration**									.10
		>3 months	4		62		0.81 (0.37-1.24)			
		≤3 months	8		0		0.41 (0.23-0.60)			
	**Frequency**									.39
		≥3 days per week	6		27		0.46 (0.21-0.71)			
		<3 days per week	6		52		0.64 (0.33-0.95)			
	**Time**									.96
		≥1 h per session	8		47		0.54 (0.26-0.82)			
		<1 h per session	4		32		0.55 (0.27-0.83)			
	**Comparator characteristics**									.69
		Active control	8		54		0.52 (0.21-0.83)			
		Inactive control	4		0		0.59 (0.39-0.80)			

^a^N/A: not applicable.

^b^Cutoff chosen was the year this updated review added newly published studies compared with the latest published review [30].

#### Domain-Specific Cognition

##### Executive Function

**Figure 3:**
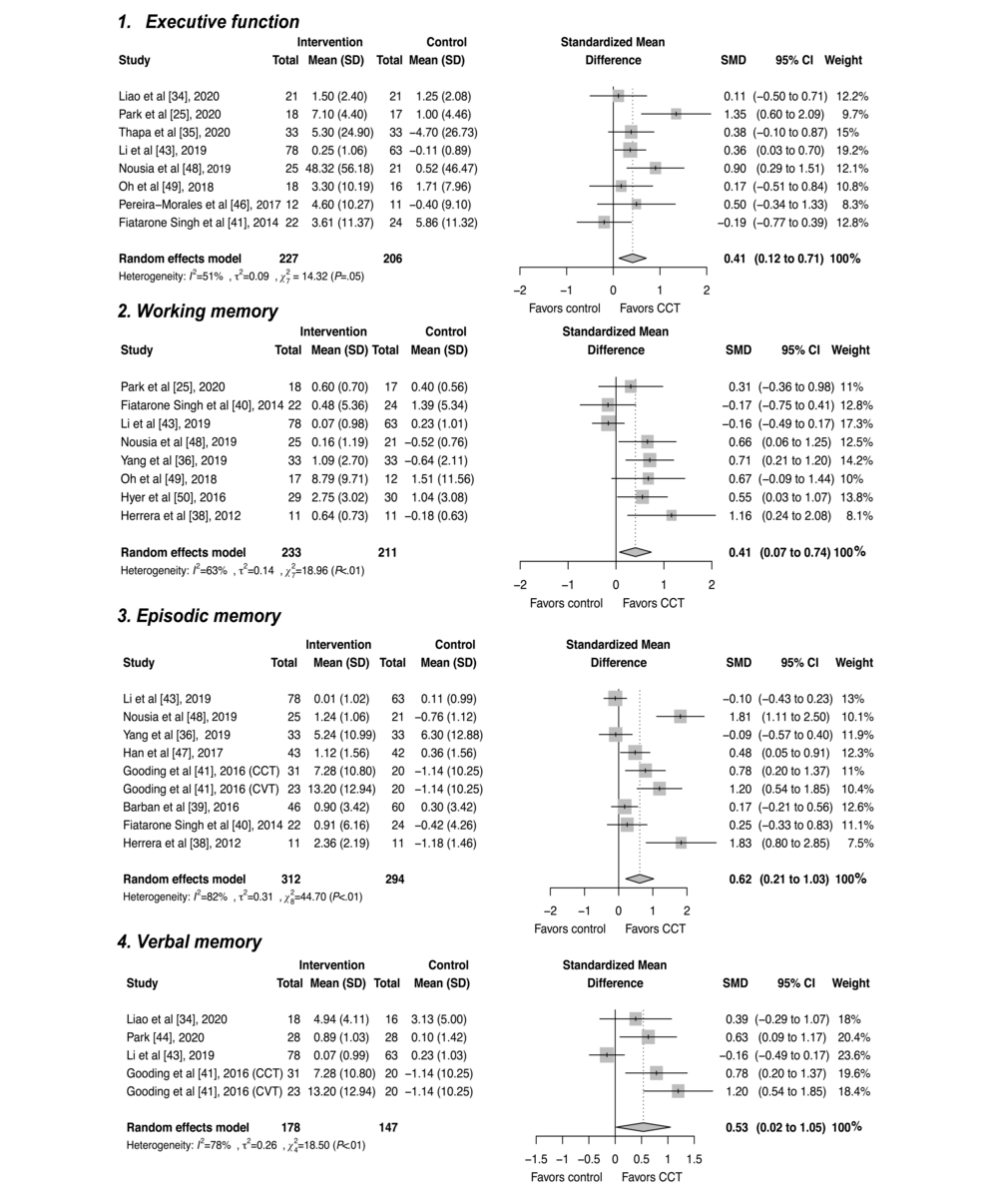
Forest plot for studies assessing specific domains of cognitive function (executive function, working memory, episodic memory, and verbal memory) [25,34-36,38-41,43,44,46-50]. CCT: computerized cognitive training; CVT: cognitive vitality training; SMD: standardized mean difference.

Eight studies assessed the change of executive function by using the Stroop test [43,46], the Trail Making Test [25,35,48], Controlled Oral Word Association Test [40], Memory Diagnostic System (executive subscale) [49], and Executive Interview [34]. As shown in Figure 3, the overall pooled SMD of executive function was 0.41 (95% CI 0.12-0.71), with moderate inconsistency between the studies (*P*=.046; *I*^2^=51%), but no publication bias was presented (Multimedia Appendix 2). The sensitivity analysis provided results consistent with the original result (Multimedia Appendix 3).

##### Working Memory

A total of 8 studies measured the change in the working memory. The digit span test was the most common instrument used to measure this outcome [25,36,38,48], followed by Auditory Logical Memory and Auditory Verbal Logical Test (immediate recall) [40,43], memory diagnostic system (working memory subscale) [49], and Span Board [50]. The working memory of the participants in the intervention groups showed an improvement compared to that of those in the control groups (SMD=0.41, 95% CI 0.07-0.74) (Figure 3). Heterogeneity across the studies was moderate (*P*=.008; *I^2^*=63%) (Multimedia Appendix 2).

##### Episodic Memory

A total of 8 studies measured the change in episodic memory by varied delayed memory recall tests [36,38-41,43,47,48]. The forest plot for episodic memory is presented in Figure 3, with a pooled SMD of 0.62 (95% CI 0.21-1.03), reflecting the benefit from the intervention group. However, the heterogeneity analyses suggested considerable heterogeneity between the studies (*P*<.001; *I^2^*=82%) (Multimedia Appendix 2).

##### Verbal and Visual Memory

Four studies specifically investigated the change in participants’ verbal memory [34,41,43,44] and revealed an SMD of 0.53 (95% CI 0.02-1.05; *P*=.001; *I^2^*=78%) in favor of CCT groups (Figure 3). No publication bias was detected in the funnel plot (Multimedia Appendix 2). In terms of the visual memory, only 1 study measured visual memory based on the Wechsler Memory Scale-Revised Visual Reproduction subset [41], but there was no significant difference between the CCT group and control (SMD=0.33, 95% CI –0.08 to 0.75).

### Other Findings

#### Adverse Effects

There were no adverse events reported from the CCT interventions across the 18 studies. However, the study conducted by Fiatarone Singh et al [40] revealed 2 adverse events in the control groups due to falls or pre-existing arthritis symptoms exacerbated while participating in strength testing or training.

#### Effect Durability and Feasibility

Five studies reported additional assessments after the end of interventions [38-40,43,50]. The duration of the follow‐up after the end of the interventions ranged from 3 to 12 months. All 5 studies evaluated the long-term maintenance of CCT-related cognitive benefits. Out of the various cognitive measures, all reported some sustained improvement, significantly better than controls. Notably, only 1 study reported dementia incidence after the training [43]. Three of the total 78 patients in the CCT group were diagnosed with Alzheimer disease in 6 months and another 3 (33 assessed) developed Alzheimer disease over 12 months after cessation of training, compared with 15 out of 63 and 6 out of 30 in the control group, respectively. No study measured participants’ satisfaction pertaining to the intervention itself. However, improved overall memory satisfaction and psychosocial satisfaction were reported [40,49].

### ROB With Studies

**Figure 4:**
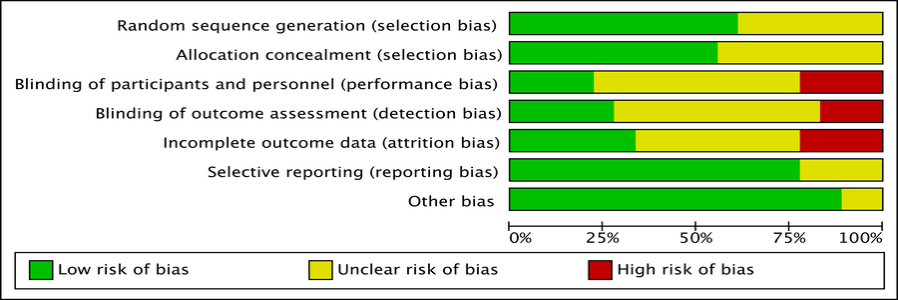
Results of risk of bias presented as percentages across all included studies.

**Figure 5:**
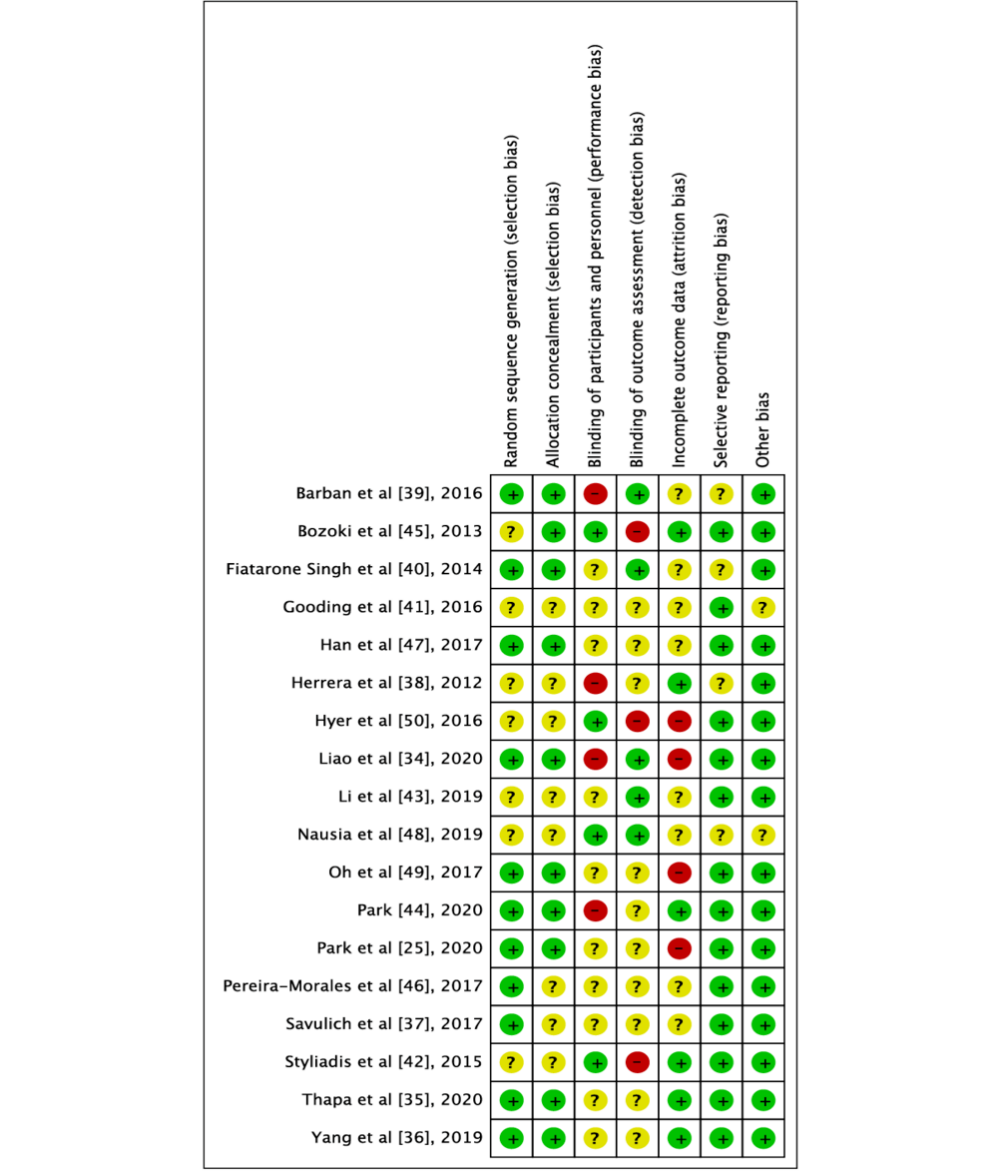
Results of risk of bias for each included study [25,34-50]. Key for colors: Red: high ROB; Yellow: unclear ROB; Green: low ROB. ROB: risk of bias.

As depicted in Figure 4 and Figure 5, no study exhibited a low ROB in all items of assessment, while 9 studies had a high ROB in at least one item of assessment [25,34,38,39,42,44,45,49,50]. Overall, 11 studies were assessed as low risk of selection bias [25,34-37,39,40,44,46,47,49], and another 7 studies [38,41-43,45,48,50] were assessed as unclear because they did not report a clear process of generation of a randomized sequence. Four studies had a high risk of performance bias, as participants were unmasked during the treatment [34,38,39,44]. The risk of detection bias was high in 3 studies [42,45,50], as outcome assessors were not blinded to the intervention allocation. Other biases were judged as low risk in all 18 studies.

## Discussion

### Summary of the Principal Findings

This systematic review synthesized 18 RCTs with a total of 1059 participants to assess the effectiveness of CCT in delaying the progression of MCI. The findings of this review indicated that CCT interventions provided a statistically significant improvement in global cognitive function. In addition, CCT interventions resulted in a positive effect in executive function, working memory, episodic memory, and verbal memory in people with cognitive decline compared to those in the control groups. We analyzed the relationship between the characteristics of CCT interventions and cognition-related health outcomes by using meta-analyses. Our results emphasized that CCT is a promising approach for improving global cognitive function. According to the subgroup analyses, more effective interventions were those that were performed within patients’ groups, which used interaction and feedback between providers and patients, and those targeting multidomain cognitive functions with longer durations per course and longer sessions, although the effect size is marginal and is not statistically significant. Interestingly, although not reaching a level of significance, subgroup analysis showed that the effect sizes in studies involving CCT sessions with no more than 3 times per week appeared to be higher than those in studies involving CCT sessions more than 3 times per week. This finding is in line with a previous meta-analysis, which showed that the intensive frequency of CCT sessions resulted in worse outcomes and training fatigue [51]. Therefore, future research should include variations in frequency of CCT delivery to assess the impact of the different treatment doses of CCT and to determine the frequency of the sessions with optimal outcomes. Although we categorized studies by all essential characteristics of CCT interventions mentioned from previous reviews, we did not identify any specific characteristics that could improve the effect of the CCT in global function. Possible explanations include the strict inclusion criteria, which meant there were a small number of studies in each subgroup as well as marked heterogeneity in study design, and the effect might be confounded by other factors that were not identified.

We found that those interventions conducted after 2019 [25,34-36] were more likely to deliver CCT by VR. VR-based training could help to overcome the barriers of lack of infrastructure, enhance motivation, and increase user participation by resembling real-life scenarios [52]. In addition, physical activities were often added to CCT interventions, especially in VR-delivered cognitive sessions. In future studies, it would be important to investigate the beneficial or synergistic effects of the combination of cognitive and physical components, especially for using such applications among older adults with MCI [53]. No study on the cost-effectiveness of CCT of MCI has been conducted. However, economic analysis is necessary for further research, especially given the huge economic burden of dementia for the society and family. Compared with traditional cognitive training, CCT is largely web-based, facilitating dissemination, and not requiring highly trained cognitive trainers (as does traditional cognitive training), and this considerably reduces the cost for patients and health systems.

### Limitations of This Study

The chief weakness of this review is the small number of studies included, especially at the level of subgroup. Second, this systematic review only included English language studies published in peer-reviewed journals, thereby potentially reducing the diversity of studies. Third, the mean age range of the participants in our studies was 68-76 years. Petersen et al [5] found that the risk of older people aged 80-84 years developing MCI is almost 4 times higher than that of those aged 60-64 years; therefore, we may lack data on the age group that might most benefit from the interventions.

### Implications for Further Study

Our review clearly shows that the quality of evidence is overall low, with small sample sizes, short follow-up duration, and imbalanced number of studies with different CCT characteristics, especially at the subgroup level. Although we show overall statistical significance, clinical significance is still questionable, and there is insufficient evidence to support the scale-up of such treatments. Several suggestions to improve the quality of trials are as follows.

First, longer term follow-up is needed. Only 4 studies conducted follow-up assessments after the end of the interventions [38-40,51]. The number of participants who develop dementia during the follow-up should also be an outcome measure in further studies. Further, concerning the problem of study design, the sample size of the included studies was small, ranging from 22 to 141. In addition, some intervention and control activities were similar, and this might have counteracted the effect of the CCT. Although computerized programs allow cognitive training designed to target specific cognitive capabilities, the problem of transfer of effects to tasks and cognitive domains not directly trained is a major issue in CCT [54]. Therefore, future research should clearly differentiate CCT interventions and control groups and identify the effectiveness of specific cognitive capacities. Further, more studies call for comprehensive analyses of the effectiveness of dual-task approaches such as cognitive training accompanied with physical activities.

Second, concerning statistical analysis, no power calculation was conducted in the included studies. It is important for studies to present sample size calculations to improve the validity of the results [55]. In addition, if the achieved smaller size differs from the planned sample size, the limitations for the implications need to be addressed.

Third, to date, there are no well-established CCT treatment guidelines. Most of the activities in the interventions were designed without standard criteria, including technical details, feasibility, and sustainability of the intervention strategies. The evidence in this review is heterogeneous in quality, completeness, and objectivity of the reporting of CCT interventions, thus making comparisons across intervention activities difficult. This is partly attributable to the multidisciplinary nature of CCT, which combines different approaches from the fields of health care and technology. The rapid pace of CCT development often outpaces the research ability to generate evidence. Therefore, a set of standards is needed, which can harmonize and improve the quality of CCT intervention, both for implementation and evidence dissemination.

### Conclusions

With aging populations increasing globally, there is a huge interest in interventions to delay or prevent cognitive decline. The findings from this review suggest that CCT may be a promising approach to improve global cognitive function and executive function. High accessibility and no necessity for delivery by trained experts are the major advantages of CCT as a clinical tool. However, studies with rational sample sizes, long-term treatment, and sufficient follow-up duration are needed to provide the evidence for recommendations for integration into clinical practice.

## Appendix

Multimedia Appendix 1 Search strategy and additional study characteristics [25,34-50].

Multimedia Appendix 2 Funnel plot.

Multimedia Appendix 3 Sensitivity analyses [25,34-37,39-43,46-49].

## Abbreviations

CCT: computerized cognitive training
MCI: mild cognitive impairment
PICO: Population, Intervention, Comparison, and Outcomes
PRISMA: Preferred Reporting Items for Systematic Reviews and Meta-Analyses
PROSPERO: Prospective Register of Systematic Reviews
RCT: randomized controlled trial
ROB: risk of bias
SMD: standardized mean difference
VR: virtual reality

## Data Availability

All data analyzed during this study are from published papers in web-based databases and included in this paper. The data sets generated during and analyzed in our study are available from the corresponding author on reasonable request.
