# Supplementary material for: Effectiveness of Computerized Cognitive Training in Delaying Cognitive Function Decline in People With Mild Cognitive Impairment: Systematic Review and Meta-analysis
Source: J Med Internet Res. 2022 Oct 27;24(10):e38624. doi: 10.2196/38624 (PMC9650579; doi:10.2196/38624)
Supplement: Multimedia Appendix 1 [file jmir_v24i10e38624_app1.docx]

## Multimedia Appendix 1. Search strategy.

**MEDLINE**

((exp mild cognitive impairment) OR ((cognit* or memory*) adj3 (func* or declin* or reduc* or disorder)).ti,ab.OR (mild neurocognitive disorder.ti,ab.) )AND ((cognitive adj3 stimulat*).ab,ti. OR (cognitive adj3 train*).ab,ti. OR (cognitive adj3 exercis*).ab,ti. OR cognitive intervention*.ab,ti. OR (cognitive adj3 rehab*).ab,ti. OR brain train*.ab,ti. OR brain exercis*.ab,ti. OR memory rehab*.ab,ti. OR memory train*.ab,ti. OR brain stimulat*.ab,ti. ) AND robot*.ab,ti. OR tablet*.ab,ti. OR phone*.ab,ti. OR ipad*.ab,ti. OR laptop*.ab,ti. OR virtual reality*.ab,ti. OR augmented reality*.ab,ti. OR video*.ab,ti. OR comput*.ab,ti. OR software*.ab,ti. OR exp technology/ limit 28 to (adaptive clinical trial or clinical study or clinical trial or controlled clinical trial or multicenter study or pragmatic clinical trial or randomized controlled trial) AND limit to english language AND limit 30 to yr="2010 - 2021"

**Web of Science**

1. TS= ("mild cognitive impairment" or "cognitive dysfunction" or ((cognit* or memory*) adj3 (func* or declin* or reduc* or disorder))))

Timespan: 2010 to 2021

Language: English

2. TS= ("cognitive stimulation" or "cognitive adj3 train*" or "cognitive intervention*" or "cognitive exercis*" or "brain train*" or "brain exercis*" or "memory rehab*" or "memory train*")

Timespan: 2010 to 2021

Language: English

3. TS= (techn* or robot* or tablet*or "smart phone*" or "computerized-software*" or "assistive technology*" or "ipad*" or "laptop*" or "virtual reality*" or VR or "artificial intelligence*" or "Augmented Reality*" or AR or video* or comput* or software*)

Timespan: 2010 to 2021

Language: English

4. TS= ("randomized controlled trial*" or RCT or random* or "controlled clinical trial")

Timespan: 2010 to 2021

Language: English

5. #4 AND #3 AND #2 AND #1

6. TS= (effect* or cost* or econom* or impact*)

Timespan: 2010 to 2021

Language: English

7. #6 AND #5

**Embase**

<2010 to 2021 Week 37>

1 exp mild cognitive impairment/

2 Cognitive Dysfunction*.ab,ti.

3 ((cognit* or memory*) adj3 (func* or declin* or reduc* or disorder)).ti,ab.

4 1 or 2 or 3

5 cognitive stimulation.ab,ti.

6 (cognitive adj3 train*).ab,ti.

7 cognitive intervention*.ab,ti.

8 cognitive exercis*.ab,ti.

9 brain train*.ab,ti.

10 brain exercis*.ab,ti.

11 memory rehab*.ab,ti.

12 memory train*.ab,ti.

13 5 or 6 or 7 or 8 or 9 or 10 or 11 or 12

14 exp technology/

15 robot*.ab,ti.

16 tablet*.ab,ti.

17 smart phone*.ab,ti.

18 computerized-software*.ab,ti.

19 assistive technology*.ab,ti.

20 ipad*.ab,ti.

21 laptop*.ab,ti.

22 virtual reality*.ab,ti.

23 artificial intelligence*.ab,ti.

24 Augmented Reality*.ab,ti.

25 video*.ab,ti.

26 comput*.ab,ti.

27 software*.ab,ti.

28 14 or 15 or 16 or 17 or 18 or 19 or 20 or 21 or 22 or 23 or 24 or 25 or 26 or 27

29 randomized controlled trial*.ab,pt,ti.

30 RCT.ab,pt,ti.

31 random*.ab,ti.

32 controlled clinical trial.ab,ti.

33 29 or 30 or 31 or 32

34 effect*.ab,ti.

35 cost*.ab,ti.

36 econom*.ab,ti.

37 impact*.ab,ti.

38 34 or 35 or 36 or 37

39 4 and 13 and 28 and 33

40 38 and 39

**Cochrane library**

#1 MeSH descriptor: [Cognitive Dysfunction] this term only

#2 (((cognit* or memory*) and (func* or declin* or reduc* or disorder))):ti,ab,kw (Word variations have been searched)

#3 #1 or #2

#4 (cognitive stimulation):ti,ab,kw OR ((cognit* or brain or memory) adj3 (train* or exercis* or rehab* or gam*)):ti,ab,kw (Word variations have been searched)

#5 MeSH descriptor: [Technology] this term only

#6 (video*):ti,ab,kw OR (comput*):ti,ab,kw OR (software*):ti,ab,kw (Word variations have been searched)

#7 (robot*):ti,ab,kw OR (tablet*):ti,ab,kw OR (smart phone*):ti,ab,kw OR (ipad*):ti,ab,kw OR (laptop*):ti,ab,kw (Word variations have been searched)

#8 (virtual reality*):ti,ab,kw OR (artificial intelligence*):ti,ab,kw OR (Augmented Reality*):ti,ab,kw (Word variations have been searched)

#9 #5 or #6 or #7 or #8

#10 (randomized controlled trial*):pt OR (RCT):ti,ab,kw OR (random*):ti,ab,kw OR (controlled clinical trial):pt (Word variations have been searched)

#11 #3 and #4 and #9 and #10 in Trials

#12 (effect*):ti,ab,kw OR (impact*):ti,ab,kw OR (cost*):ti,ab,kw OR (econom*):ti,ab,kw (Word variations have been searched)

#13 #11 and #12 in Trials

**Google Scholar**

(cognitive impairment* OR cognitive decline* OR cognitive dysfunction*) AND (cognitive training* OR brain training*) AND (computer* OR virtual* OR smart phone* OR digital*)

## Multimedia Appendix 1. Studies characteristics.

| **Study** | **Country** | **Setting** | **Training dose: duration; frequency; time per session** | **Cognition measurements** |
| --- | --- | --- | --- | --- |
| Liao et al [34], 2020 | Taiwan (China) | individual | 3 months; 3 day/week; 60 min | MoCA; The executive interview 25; Verbal Learning Test |
| Thapa et al [35], 2020 | Korea | individual | 2 months; 3 days/ week; 100 min | MMSE; Trail Making Test A&B; symbol digit substitution tests |
| Park [44], 2020 | Korea | individual | 2 months; 3 days/ week; 45 min | MMSE; Weschsler Adult Intelligence Scale-Revised Block Design Test; Seoul Verbal Learning Test |
| Park et al [25], 2020 | Korea | supervised | 1.5 months; 5 days/ week; 30 min | MoCA; Trail Making Test A&B; Digit Span Test-forward & backward |
| Li et al [43], 2019 | China | individual | 6 months; 3-4 days/week; 40 min | MMSE; Addenbrooke’s cognitive examination-revised; the auditory verbal tests-Huashan version; the shape trail test; the Rey-Osterrieth complex figure test; symbol digit substitution test; the Stoop Color-Word Test |
| Nousia et al [48], 2019 | Greece | individual | 3.5 month; 2 sessions/week; 60 min | MoCA; Clock Drawing Test; immediate word recall., word recognition and delayed word memory test; Boston Naming Test; Semantic Fluency measure; digit span forward; Trail-making Test A&B |
| Yang et al [36], 2019 | Taiwan (China) | individual | 3 months; 3 days/week; 45 min | MMSE; MoCA; Digit span backward; Wechsler Memory Scale; immediate memory index; delayed memory index; Multifaced Memory Questionnaire |
| Oh et al [49], 2018 | Korea | individual session + within groups | 2 months; 5 days/week; 20 min | MMSE; Wechsler Adult Intelligence Scale; The memory Diagnostic Symptom; Stroop Color and Word Test |
| Pereira-Morales et al [46], 2017 | Colombia | individual | 2 months; 4 days/week; 60 min | MMSE; the Grober and Buschke test; attention and memory subscales of the Wechsler Adult Intelligence Scale; Clock Drawing Test; Trail Making Test A&B |
| Savulich et al [37], 2017 | UK | supervised | 1 month; 2 sessions/week; 60 min | MMSE；Cambridge Neuropsychological Test Automated Battery Paired Associates Learning; Brief Visuospatial Memory Test-Revised |
| Han et al [47], 2017 | Korea | individual | 1 month; 2 days/week; 30 min | Categorical Fluency Test; the Modified Boston Naming Test, MMSE, the Word List Memory Test; the Constructional Praxis Test, the Word List Recall Test; the Word List Recognition Test; the Constructional Recall Test; the Trail Making Test A; Digit Span Test |
| Hyer et al [50], 2016 | US | individual | 5-7 week; 5 session/ week; 40 min | Wechsler Memory Scale; Span Board subtest; Trail Making Test A&B; Wechsler Adult Intelligence Scale |
| Gooding et al [41], 2016 | US | individual | 4 months; 2 sessions /week; 60 min | MMSE; Wechsler Adult Intelligence Scale- Revised Digit Span; Buschke Selective Test; Wechsler Memory Scale-Revised Logical Memory or Buschke Selective Reminding Test |
| Barban et al [39], 2016 | Italy, Greece, Norway and Spain | group and supervised | 3 months; 2 days/week; 60 min | MMSE; Rey Auditory Verbal Learning Test; Rey-Osterrieth Complex Figure Test; Trail Making Test; the Phonological Verbal Fluency Test |
| Styliadis et al [42], 2015 | Greece | individual | 2 months; 3-5 days/week; 60 min | MMSE |
| Fiatarone-Singh et al [40], 2014 | Australia | supervised | 6 months; 2 days/week; 60-100 min | ADAS-Cog; Trail Making Test A&B; Wechsler Adult Intelligence Scale; Symbol Digit Modalities Test; Logical memory of the Wechsler Memory Scale; Benton Visual Retention Test |
| Bozoki et al [45], 2013 | US, Slovenia | individual | 1.5 months; 5 days/week; 30 min | CogState |
| Herrera et al [38], 2012 | France | supervised | 3 months; 2 times/week; 60 min | Digit Span Test-forward & backward; 12-word-list recall test from the BEM-144 memory battery; the 16-item free and cued remaining test; subscore recall of the MMSE; visual recognition subtest from the Doors and people memory battery |
