# Supplementary material for: Effectiveness of Computerized Cognitive Training in Delaying Cognitive Function Decline in People With Mild Cognitive Impairment: Systematic Review and Meta-analysis
Source: J Med Internet Res. 2022 Oct 27;24(10):e38624. doi: 10.2196/38624 (PMC9650579; doi:10.2196/38624)
Supplement: Multimedia Appendix 2 [file jmir_v24i10e38624_app2.docx]

## Multimedia Appendix 2. Funnel plots.

**Funnel plot of global cognitive function**

**Funnel plot of executive function**

**Funnel plot of working memory**

**Funnel plot of episodic memory**

**Funnel plot of verbal memory**
