# Supplementary material for: Effectiveness of Computerized Cognitive Training in Delaying Cognitive Function Decline in People With Mild Cognitive Impairment: Systematic Review and Meta-analysis
Source: J Med Internet Res. 2022 Oct 27;24(10):e38624. doi: 10.2196/38624 (PMC9650579; doi:10.2196/38624)

## Multimedia Appendix 3. Sensitivity analyses.

**Forest plot of sensitivity analysis (global cognitive function)**


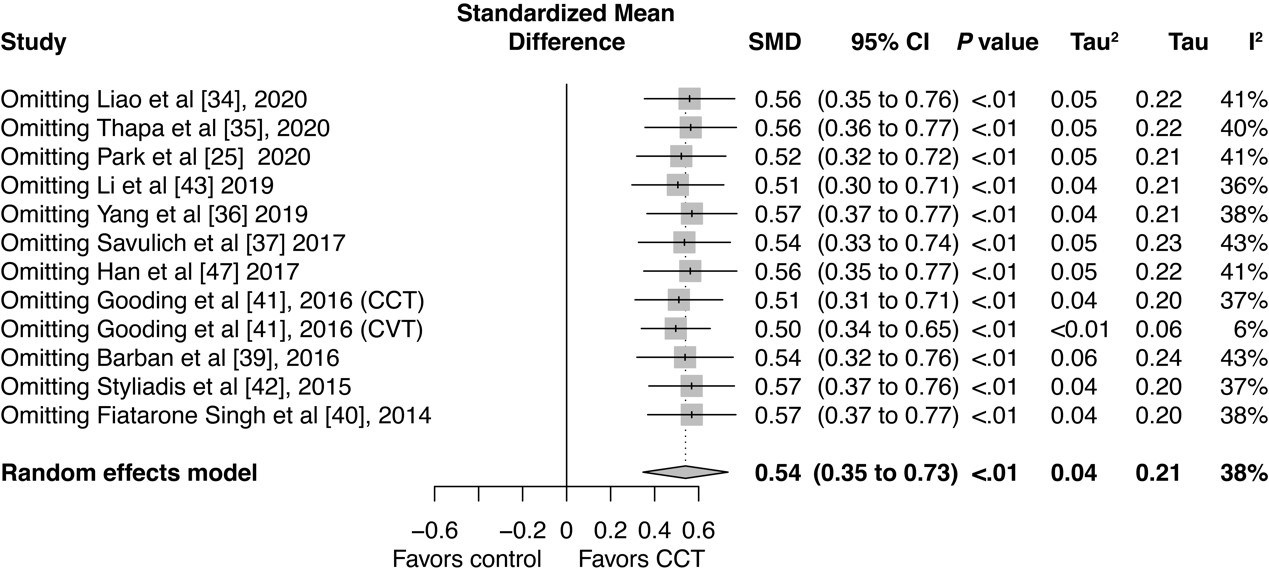


**Forest plot of sensitivity analysis (executive function)**


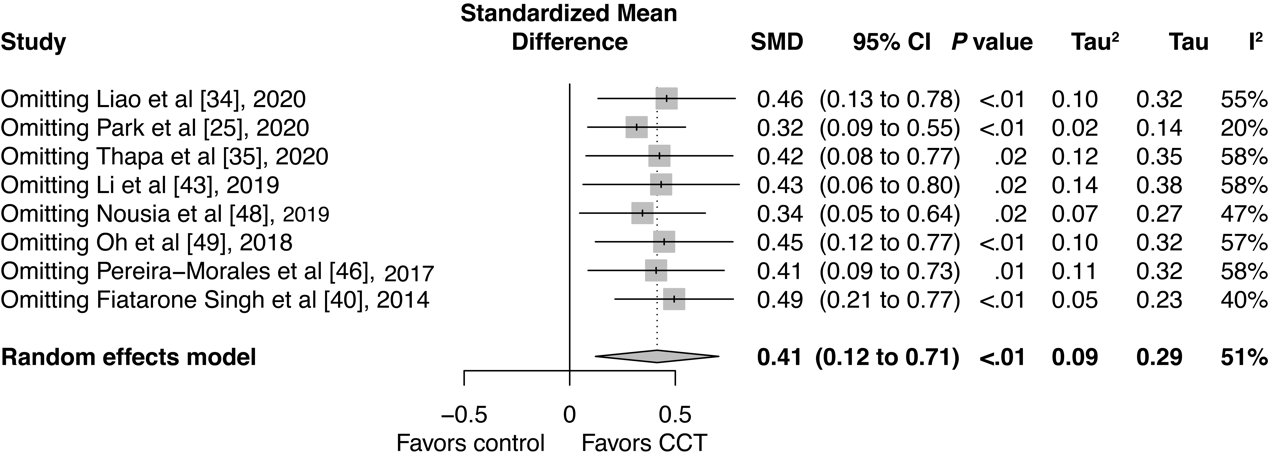

Supplement: Multimedia Appendix 3 [file jmir_v24i10e38624_app3.docx]
